# Characteristic analysis of clinical trials for new traditional Chinese medicines in mainland China from 2013 to 2021

**DOI:** 10.3389/fmed.2022.1008683

**Published:** 2022-10-18

**Authors:** Yinghong Zhou, Juan Yang, Yingchun He, Yinghua Lv, Chunli Wang, Hongyong Deng, Jihan Huang

**Affiliations:** ^1^Science and Information Center, Shanghai University of Traditional Chinese Medicine, Shanghai, China; ^2^Center for Drug Clinical Research, Institute of Interdisciplinary Integrative Medicine Research, Shanghai University of Traditional Chinese Medicine, Shanghai, China; ^3^Information Center, Shanghai Institute of Materia Medica, Chinese Academy of Sciences, Shanghai, China

**Keywords:** new Chinese medicine, clinical trial, registration application, study design, phase II trial, phase III trial

## Abstract

**Objective:**

Based on the clinical trials registered on the platform for the registry and publicity of clinical drug trials of the National Medical Products Administration (NMPA), the registration and approval of clinical trials of traditional Chinese medicines (TCMs) in mainland China from 2013 to 2021 were reviewed.

**Methods:**

Clinical trials of new TCMs published in Chinese were retrieved from the platform for the registry and publicity of clinical drug trials. The number of registered trials and approved trials, status of clinical trials, therapeutic area of clinical trials for the treatment of diseases, type of trial design, sample size, sponsors, and leading clinical trial centers were evaluated.

**Results:**

From 2013 to 2021, a total of 965 clinical trials of new drugs applied in TCM were registered on the aforementioned NMPA platform, comprising 117 phase I trials, 586 phase II trials, 174 phase III trials, 40 phase IV trials, and 48 other clinical trials. The treatment fields included the respiratory system, alimentary tract and metabolism, genetic system and reproductive hormones, and cardiovascular system. Among the 760 phase II and phase III trials, 98.9% were randomized, 95.4% were double-blind, and 98.2% were parallel controlled trials, and the proportion of placebo-controlled trials increased year by year from 2013 to 2021. From 2013 to 2021, 123 new TCMs were approved in mainland China.

**Conclusion:**

From 2015 to 2021, the number of registered clinical trials of new TCMs remained low. The approval rate was also low, but the clinical trial design was greatly improved.

## Introduction

Traditional Chinese medicine (TCM) originated in ancient China and has evolved over thousands of years (1). The development of TCM represents a valuable medical achievement, and natural medicine is regarded as nature's gift to people. During the COVID-19 pandemic, TCM has played a role in disease treatment (2, 3). Clinical trials are considered the gold standard for evaluating the safety and efficacy of therapeutics and generating evidence-based knowledge in the medical field (4). Clinical drug trials are a necessary verification procedure implemented before a new drug is approved. The data relating to the safety and efficacy of new drugs provide a valuable evaluation basis for the approval of new drugs. Clinical trials of new TCMs must fully comply with the requirements of good clinical practice (GCP) to ensure the standardized process of clinical drug trials is followed, scientific and reliable data are collected, and the rights, interests, and safety of trial participants are protected.

The seventh revision of the Declaration of Helsinki contains new requirements related to the registration of clinical trials and reporting of the results (5). Sharing individual participant data is a key approach to respecting the contributions of participants and is essential for the future of clinical trials.

On 6 September 2013, the National Medical Products Administration (NMPA) issued Announcement No. 28 on its drug clinical trial information platform calling all approved clinical drug trials to be registered and publicized on the platform for the registry and publicity of clinical drug trials. This requirement is a critical measure for strengthening the supervision and management of clinical drug trials, promoting the openness and transparency of clinical drug trial information, and protecting the rights, interests, and safety of trial participants (6).

The registration and publicity of clinical drug trials ensures the public's right to know and the rights and interests of participants and enables researchers to continually adjust and optimize their test protocols promptly and improve scientific test design through learning and studying similar clinical trial designs, test methods, and technical approaches (7).

Based on registered clinical trial data, Chinese researchers have conducted meaningful studies. For example, Dawei Wu et al. (8) summarized the progress of the clinical trials of cancer drugs in China in 2020. Ning Li et al. (9) reviewed changes in the clinical trials of cancer drugs in mainland China between 2009 and 2018. Wenwen Wu et al. (10) analyzed pediatric clinical trials in mainland China over the past decade, and Qiaofeng Zhong et al. (11) reviewed the changing landscape of anti-lung-cancer clinical drug trials in mainland China from 2005 to 2020.

Using the aforementioned studies as our foundation, we reviewed the registration and approval of clinical trials of new TCM drugs from 2013 to 2021, including the trial design, treatment field, and distribution of application units and team leaders. Our research provides basic data to assist sponsors and researchers in conducting research on and developing TCMs and can serve as a reference for improving the quality and efficiency of the research and development of new TCMs.

## Materials and methods

### Data source

The platform for the registry and publicity of clinical drug trials was established by the NMPA's Center for Drug Evaluation (www.chinadrugtrials.org.cn). In the advanced search options, we chose “Chinese medicine/natural medicine” as the keyword in drug type. Researchers who have obtained approval from the NMPA to conduct clinical trials in China (including bioequivalence, pharmacokinetics [PK], and phase I–IV trials) must register their trials and publicize the trial information on this platform.

### Search strategy

We searched for clinical trials involving TCMs or natural medicines that were conducted from January 2013 to December 2021. We then downloaded all retrieved records and collated each trial registration number, trial name, indicator, drug name, drug type, trial classification, trial stage, design type (e.g., randomized and blind trial), trial scope, date of first publicized information, date of enrollment of the first trial participant, leading unit, participating unit, sponsor, and source of funds of the test project.

### Statistical analysis

All data analyses were performed using SAS version 9.4 (SAS, Cary, NC, USA). Frequencies and percentages were used to describe categorical variables.

## Results

### Number of new drugs trials registered

**Figure 1:**
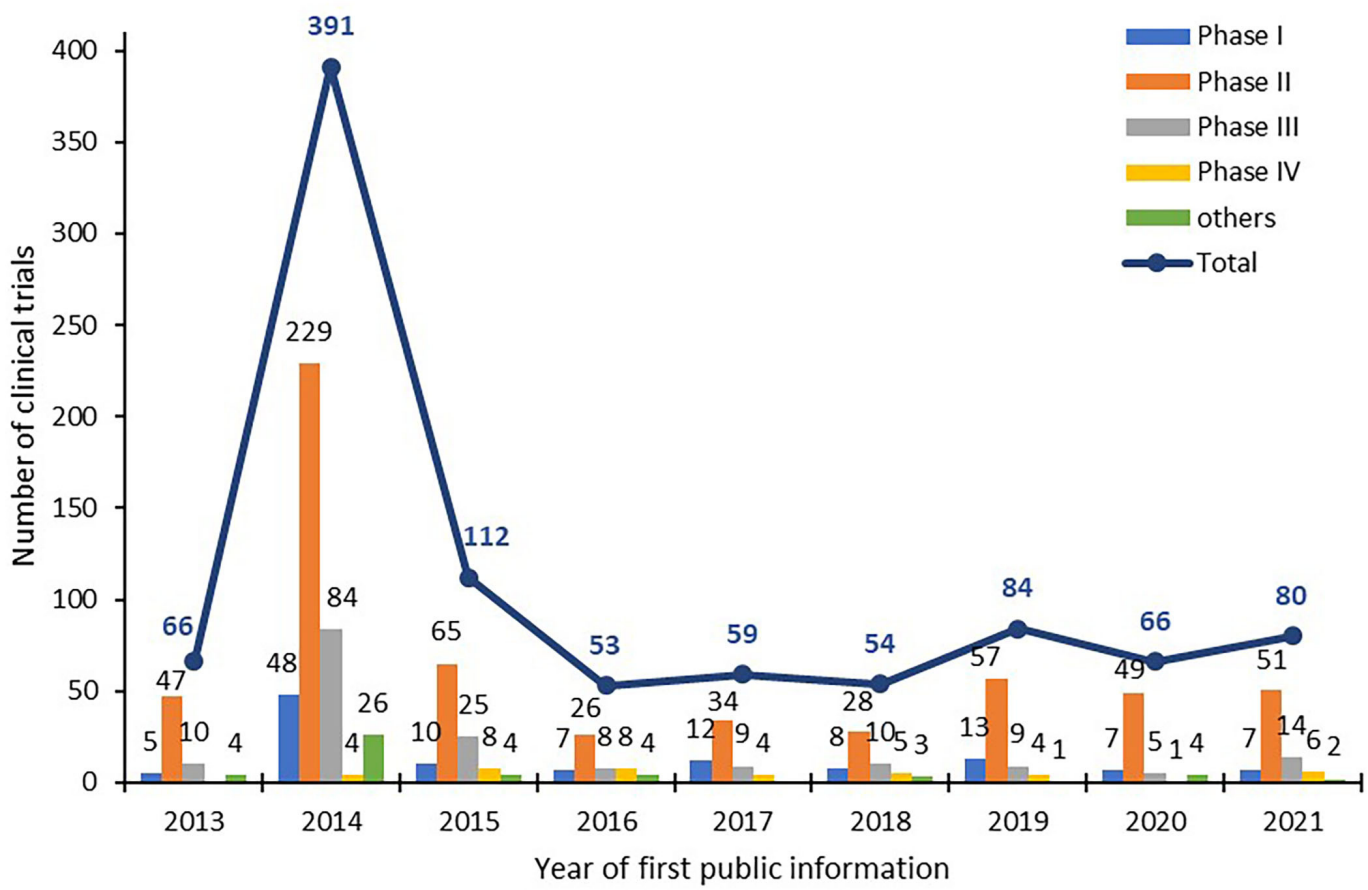
Number of trials of new TCMs registered in mainland China.

From 2013 to 2021, 965 clinical trials of new TCMs were registered on the platform for the registry and publicity of clinical drug trials in mainland China. A total of 66, 391, 112, 53, 59, 54, 84, 66, and 80 items were registered each year from 2013 to 2021 (according to the “date of first publicized information”). The number of clinical trials registered in 2014 was much higher (391) than that in other years because the NMPA platform was launched in 2013 and applicant institutions were required to register trials initiated before 2014 retrospectively. After the large number of submissions in 2013 and 2014, the number of annually submitted trials remained between 50 and 100 from 2015 to 2021, as illustrated in Figure 1.

### Clinical trial status and trial phase

**Figure 2:**
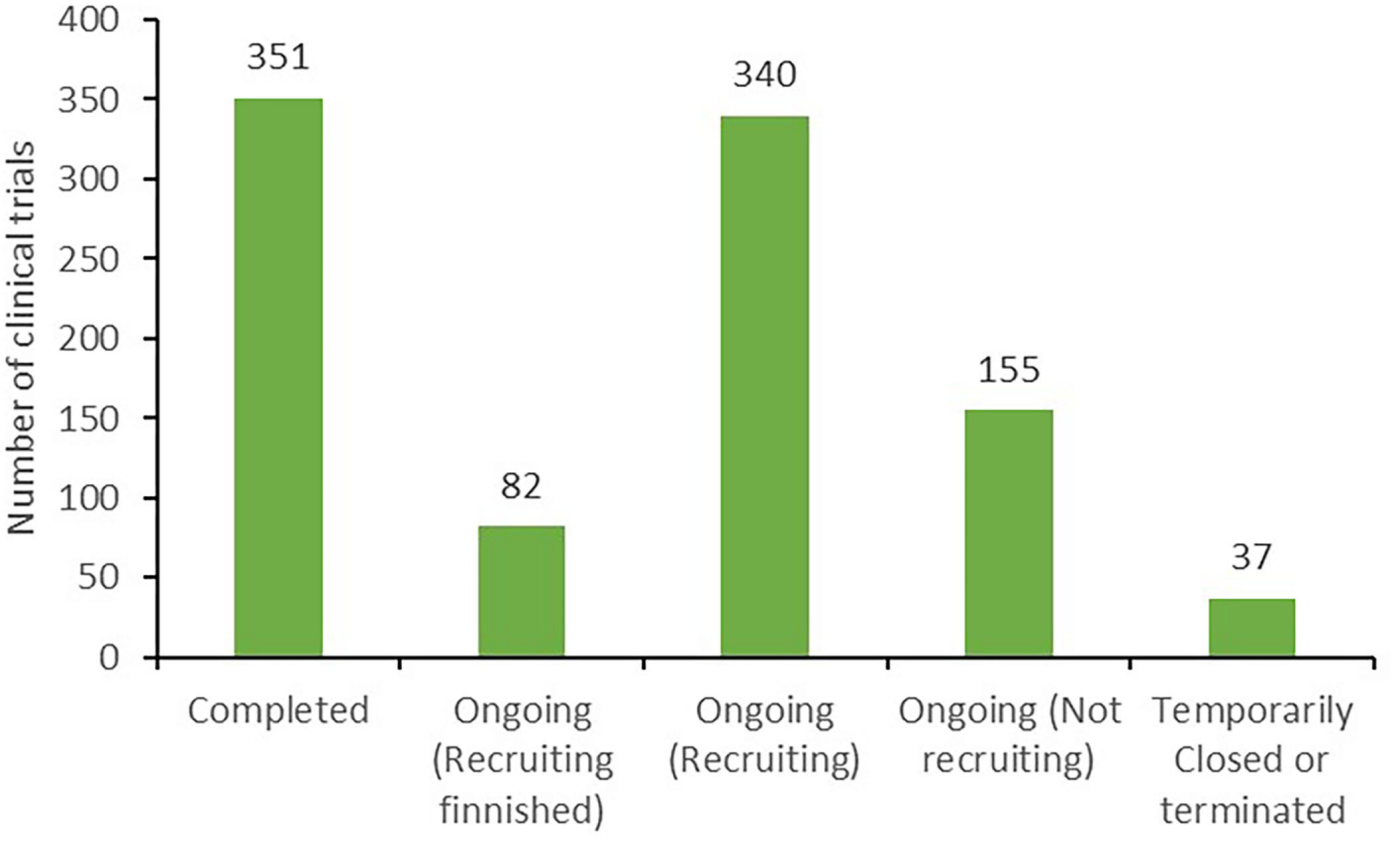
Classification of the status of clinical trials.

According to clinical trial status, all 965 clinical trials could be categorized as either completed trials (351, 36.4%); those in which recruitment was complete (82, 8.5%), ongoing (340, 35.2%), or had not yet begun (155, 16.1%); or temporarily halted or terminated trials (37, 3.8%), as presented in Figure 2.

**Figure 3:**
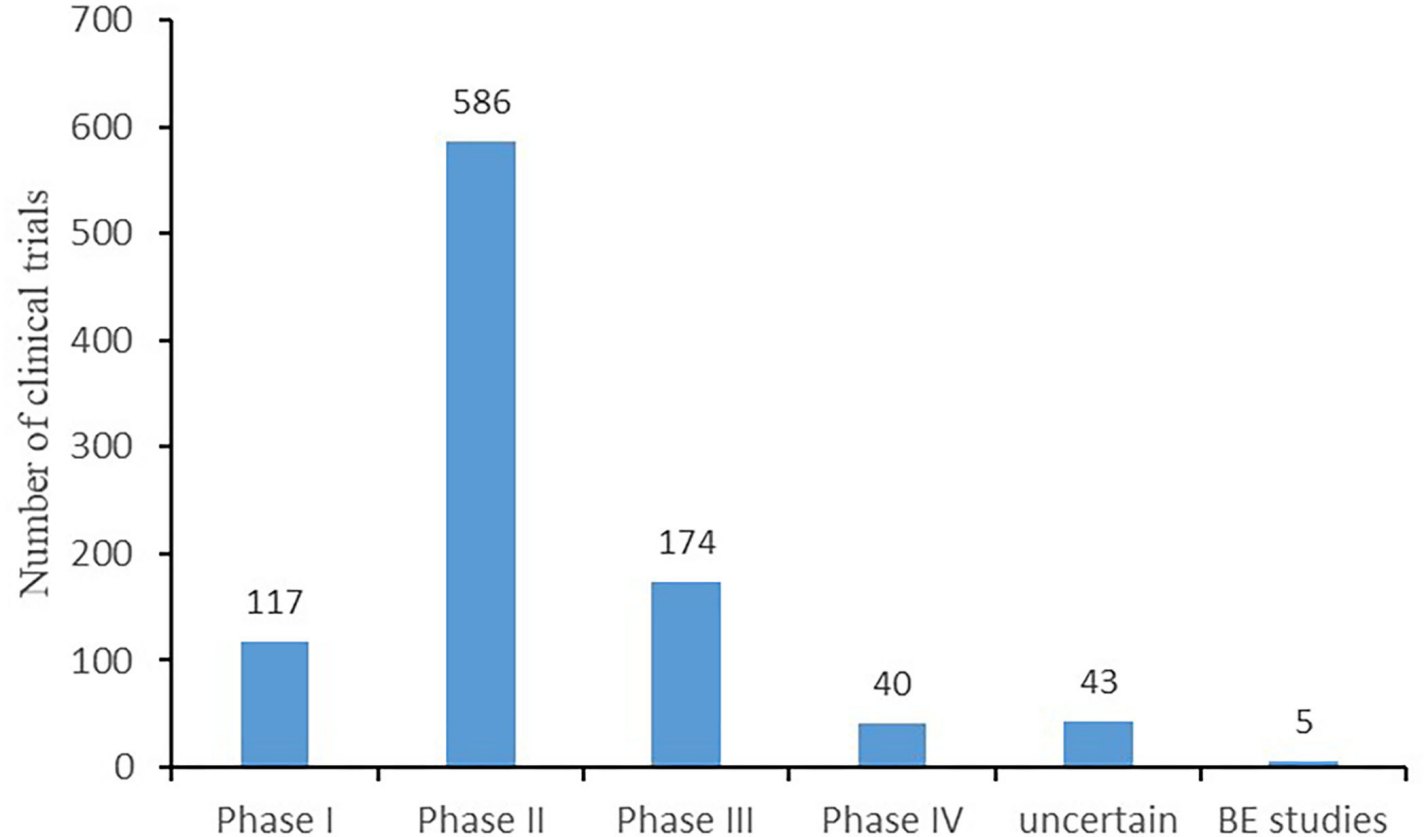
Phases of clinical trials.

Regarding the phase distribution of the 965 trials, phase II trials accounted for the largest proportion (586, 60.7%), followed by phase III trials (174, 18%), phase I trials (117, 12.1%), trials of uncertain phase (43, 4.5%), and phase IV trials (40, 4.1%). The rest (5, 0.5%) were BE studies, as depicted in Figure 3.

### Study design of phase II to IV trials

With regard to allocation type, randomized clinical trials accounted for 95.3% (762) of all trials and non-randomized for 4.8% (38).

Concerning the intervention model, parallel assignment accounted for 94.1% (753) of all trials, factorial assignment for 0.3% (2), single-group assignment for 5.4% (43), and crossover assignment for 0.3% (2).

Regarding blinding settings, 91.1% (729) of all trials were double-blind, 1.6% (13) were single-blind, and 7.3% (58) were open labels. In terms of geographical scope, 99.6% (797) of the trials were domestic multicenter or single-center studies and 0.4% (3) were international multicenter studies.

When classified according to control assignment, 66.1% (529) of the trials were placebo control only, 15.5% (124) were positive control only, 9.4% (75) were double placebo and positive control, and the rest 9% (72) were trials with no control group.

Regarding the number of groups, trials that involved one group accounted for 5.0% (40), and those with two and three groups accounted for 47.4% (379) and 39% (312), respectively. The remaining 8.6% (69) involved four or more groups.

Of the 800 trials, 724 had specific requirements for sample size, which ranged between 15 and 6,600 individuals, with a cumulative statistical sample size of 249,960 participants. Moreover, a minimum sample size of 15 individuals was used in trial CTR20140903, which was sponsored by Jiangsu Kangyuan Pharmaceutical and China Pharmaceutical University and executed by The First Affiliated Hospital of Nanjing Medical University. Moreover, sample sizes larger than 3,000 were used in three clinical trials, namely CTR20132020, CTR20160479, and CTR20140108; all these trials were phase IV trials. As summarized in Table 1, 8, 19, 38.4, 3.9, 11.3, 6.3, 3.3, and 0.4 of the trials involved 100 or less, 101–200, 201–300, 301–400, 401–500, 501–1,000, 1,001–3,000, and more than 3,000 participants, respectively.

**Table 1:** Trial characteristics and designs for phase II–IV trials.

**Category**	**Information**	**Phase II(*N* = 586)**	**Phase III(*N* = 174)**	**Phase IV(*N* = 40)**	**Total (*N* = 800)**
Allocation	Randomized, *n* (%)	578(98.6%)	174(100.0%)	10(25.0%)	762(95.3)
	Not randomized, *n* (%)	8(1.4%)	0(0.0%)	30(75.0%)	38(4.8)
Intervention model	Parallel Assignment, *n* (%)	573(97.8%)	173(99.4%)	7(17.5%)	753(94.1)
	Crossover Assignment, *n* (%)	2(0.3%)	0(0.0%)	0(0.0%)	2(0.3)
	Single Group Assignment, *n* (%)	10(1.7%)	0(0.0%)	33(82.5%)	43(5.4)
	Factorial Assignment, *n* (%)	1(0.2%)	1(0.6%)	0(0.0%)	2(0.3)
Masking	Double blind, *n* (%)	557(95.1%)	168(96.6%)	4(10.0%)	729(91.1)
	Single blind, *n* (%)	8(1.4%)	4(2.3%)	1(2.5%)	13(1.6)
	Open label, *n* (%)	21(3.6%)	2(1.1%)	35(87.5%)	58(7.3)
Test scope	Domestic, *n* (%)	585(99.8%)	172(98.9%)	40(100.0%)	797(99.6)
	International Multi-center, *n* (%)	1(0.2%)	2(1.1%)	0(0.0%)	3(0.4)
Numbers of group	One group, *n* (%)	7(1.2%)	0(0.0%)	33(82.5%)	40(5.0)
	Two group, *n* (%)	235(40.1%)	139(79.9%)	5(12.5%)	379(47.4)
	Three group, *n* (%)	279(47.6%)	32(18.4%)	1(2.5%)	312(39.0)
	≥Four group, *n* (%)	65(11.1%)	3(1.7%)	1(2.5%)	69(8.6)
Control assignment	Placebo control only, *n* (%)	417(71.2%)	109(62.6%)	3(7.5%)	529(66.1)
	Positive control only, *n* (%)	90(15.4%)	31(17.8%)	3(7.5%)	124(15.5)
	Both, *n* (%)	44(7.5%)	31(17.8%)	0(0.0%)	75(9.4)
	Neither, *n* (%)	35(6.0%)	3(1.7%)	34(85.0%)	72(9.0)
Sample sizes	≤ 100 cases, *n* (%)	62(10.6%)	1(0.6%)	1(2.5%)	64(8.0)
	101–300 cases, *n* (%)	434(74.1%)	21(12.1%)	4(10.0%)	459(57.4)
	301–1000 cases, *n* (%)	30(5.1%)	134(77.0%)	7(17.5%)	171(21.4)
	>1000 cases, *n* (%)	0(0.0%)	2(1.1%)	28(70.0%)	30(3.8)
Sample sizes	Median (IQR)	226.5(144–240)	480(480–550)	767(2000–2200)	240(144–297)

A total of 60 and 18 phase II and phase III trials, respectively, did not provide a sample size.

We conducted an annual analysis of the methodological parameters of Phase II and Phase III clinical trials, such as allocation, intervention model, masking, and control assignment (Table 2). In the past 9 years, the annual changes in design features, such as allocation and intervention model, were not significant. Among them, random design and parallel design were always prevailing, accounting for no less than 97.4 and 95.3%, respectively. In contrast, the other two design features, blinding design and control assignment, showed obvious annual changes. Although the proportion of double-blind design is more than 90% every year, 91.2% of the trials adopted double-blind design in 2016, while in 2021, 100% of the trials were double-blind. The proportion of placebo-controlled trials increased year by year. From 2013 to 2021, the proportion of placebo control only or double control trials was 77.2, 74.8, 78.9, 79.4, 79.1, 81.6, 80.3, 85.2, and 93.8, respectively. The percentage of placebo-controlled trials in 2021 is 19.0% higher than that in 2014.

**Table 2:** Trial characteristics and designs for phase II–phase III trials in different years.

**Category**	**Information**	**2013** **(*N* = 57)**	**2014** **(*N* = 313)**	**2015** **(*N* = 90)**	**2016** **(*N* = 34)**	**2017** **(*N* = 43)**	**2018** **(*N* = 38)**	**2019** **(*N* = 66)**	**2020** **(*N* = 54)**	**2021** **(*N* = 65)**
Allocation	Randomized, *n* (%)	57 (100.0%)	310 (99.0%)	88 (97.8%)	34 (100.0%)	42 (97.7%)	37 (97.4%)	66 (100.0%)	53 (98.1%)	65 (100.0%)
	Not randomized, *n* (%)	0 (0.0%)	3 (1.0%)	2 (2.2%)	0 (0.0%)	1 (2.3%)	1 (2.6%)	0 (0.0%)	1 (1.9%)	0 (0.0%)
Intervention model	Parallel Assignment, *n* (%)	57 (100.0%)	306 (97.8%)	88 (97.8%)	33 (97.1%)	41 (95.3%)	37 (97.4%)	66 (100.0%)	53 (98.1%)	65 (100.0%)
	Crossover Assignment, *n* (%)	0 (0.0%)	2 (0.6%)	0 (0.0%)	0 (0.0%)	0 (0.0%)	0 (0.0%)	0 (0.0%)	0 (0.0%)	0 (0.0%)
	Single Group Assignment, *n* (%)	0 (0.0%)	4 (1.3%)	2 (2.2%)	0 (0.0%)	2 (4.7%)	1 (2.6%)	0 (0.0%)	1 (1.9%)	0 (0.0%)
	Factorial Assignment, *n* (%)	0 (0.0%)	1 (0.3%)	0 (0.0%)	1 (2.9%)	0 (0.0%)	0 (0.0%)	0 (0.0%)	0 (0.0%)	0 (0.0%)
Masking	Double blind, *n* (%)	56 (98.2%)	298 (95.2%)	86 (95.6%)	31 (91.2%)	40 (93.0%)	35 (92.1%)	62 (93.9%)	52 (96.3%)	65 (100.0%)
	Single blind, *n* (%)	1 (1.8%)	8 (2.6%)	2 (2.2%)	0 (0.0%)	0 (0.0%)	0 (0.0%)	1 (1.5%)	0 (0.0%)	0 (0.0%)
	Open label, *n* (%)	0 (0.0%)	7 (2.2%)	2 (2.2%)	3 (8.8%)	3 (7.0%)	3 (7.9%)	3 (4.5%)	2 (3.7%)	0 (0.0%)
Test scope	Domestic, *n* (%)	57 (100.0%)	313 (100.0%)	90 (100.0%)	34 (100.0%)	43 (100.0%)	37 (97.4%)	66 (100.0%)	54 (100.0%)	63 (96.9%)
	International Multi-center, *n* (%)	0 (0.0%)	0 (0.0%)	0 (0.0%)	0 (0.0%)	0 (0.0%)	1 (2.6%)	0 (0.0%)	0 (0.0%)	2 (3.1%)
Numbers of group	One group, *n* (%)	0 (0.0%)	2 (0.6%)	1 (1.1%)	0 (0.0%)	2 (4.7%)	2 (5.3%)	0 (0.0%)	0 (0.0%)	0 (0.0%)
	Two group, *n* (%)	31 (54.4%)	160 (51.1%)	44 (48.9%)	14 (41.2%)	16 (37.2%)	12 (31.6%)	34 (51.5%)	24 (44.4%)	36 (55.4%)
	Three group, *n* (%)	25 (43.9%)	128 (40.9%)	35 (38.9%)	15 (44.1%)	20 (46.5%)	18 (47.4%)	26 (39.4%)	23 (42.6%)	21 (32.3%)
	≥Four group, *n* (%)	1 (1.8%)	23 (7.3%)	10 (11.1%)	5 (14.7%)	5 (11.6%)	6 (15.8%)	6 (9.1%)	4 (7.4%)	8 (12.3%)
Control assignment	Placebo control only, *n* (%)	37 (64.9%)	198 (63.3%)	64 (71.1%)	24 (70.6%)	31 (72.1%)	22 (57.9%)	51 (77.3%)	44 (81.5%)	55 (84.6%)
	Positive control only, *n* (%)	10 (17.5%)	71 (22.7%)	13 (14.4%)	4 (11.8%)	3 (7.0%)	2 (5.3%)	11 (16.7%)	5 (9.3%)	2 (3.1%)
	Both, *n* (%)	7 (12.3%)	36 (11.5%)	7 (7.8%)	3 (8.8%)	3 (7.0%)	9 (23.7%)	2 (3.0%)	2 (3.7%)	6 (9.2%)
	Neither, *n* (%)	3 (5.3%)	8 (2.6%)	6 (6.7%)	3 (8.8%)	6 (14.0%)	5 (13.2%)	2 (3.0%)	3 (5.6%)	2 (3.1%)
Sample sizes	≤ 100 cases, *n* (%)	2 (9.5%)	21 (6.7%)	9 (10.0%)	5 (14.7%)	4 (9.3%)	5 (13.2%)	5 (7.6%)	6 (11.1%)	6 (9.5%)
	101–300 cases, *n* (%)	13 (61.9)	176 (56.2%)	52 (57.8%)	18 (52.9%)	29 (67.4%)	23 (60.5%)	54 (81.8%)	44 (81.5%)	46 (73.0%)
	301–1000 cases, *n* (%)	6 (28.6%)	78 (24.9%)	29 (32.2%)	11 (32.4%)	10 (23.3%)	10 (26.3%)	7 (10.6%)	3 (5.6%)	10 (15.9%)
	>1000 cases, *n* (%)	0 (0.0%)	0 (0.0%)	0 (0.0%)	0 (0.0%)	0 (0.0%)	0 (0.0%)	0 (0.0%)	1 (1.9%)	1 (1.6)
Sample sizes	Median (IQR)	240 (180–432)	240 (216–432)	240 (180–480)	240 (144–360)	240 (144–294)	240 (180–354)	222 (120–240)	216 (120–240)	216 (138–240)

### Therapy areas of disclosed trials

**Figure 4:**
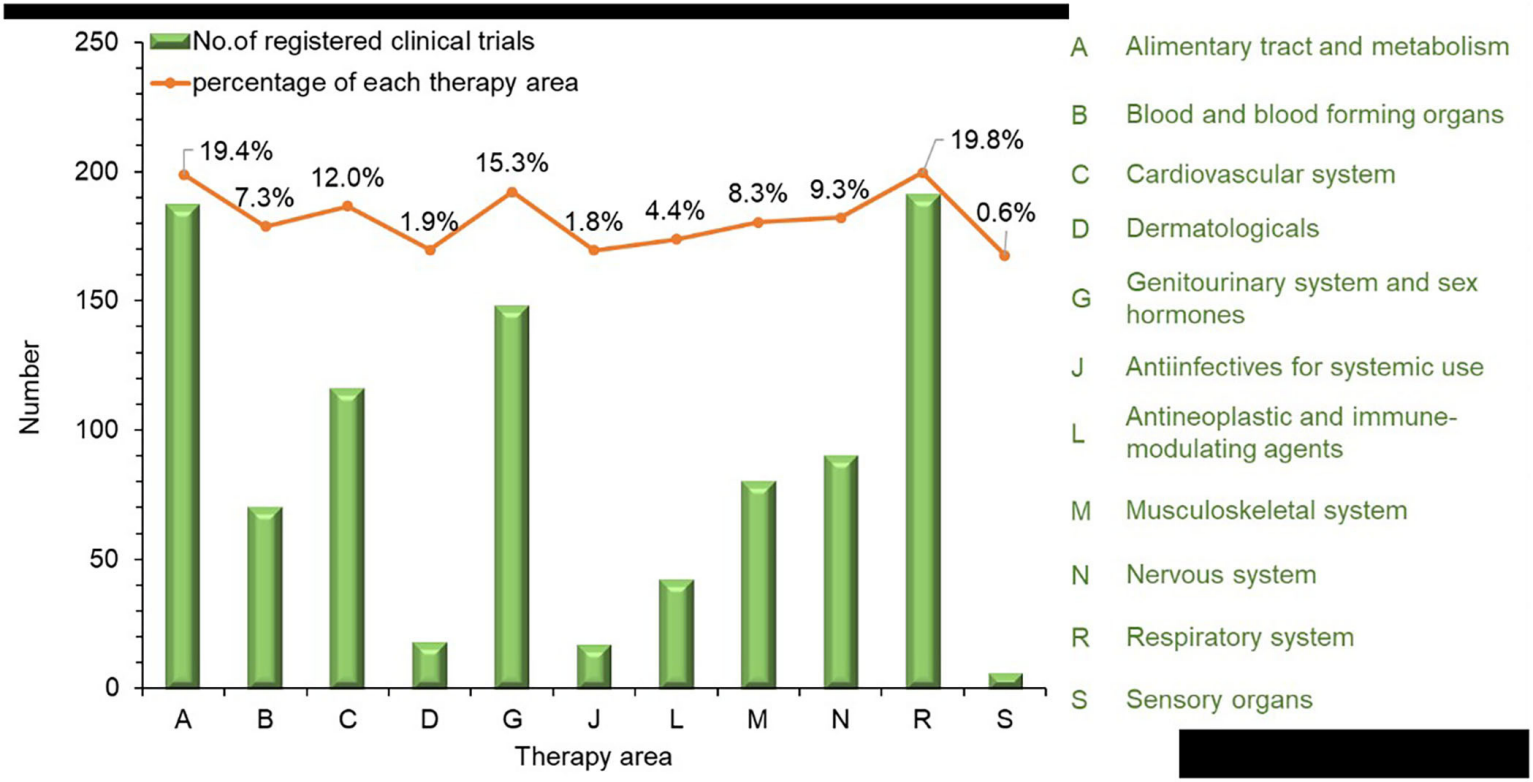
Therapy area distribution of TCM trials registered from 2013 to 2021 in mainland China.

Therapy areas were classified manually according to the Anatomical Therapeutic Chemical classification system. From 2013 to 2021, the largest number of trials involved treatments for the respiratory system at 191 (19.8%), followed by 187 (19.4%) trials for the alimentary tract and metabolism, 148 (15.3%) for the genitourinary system and reproductive hormones, and 116 (12%) for the cardiovascular system. For other therapy areas, the numbers of clinical trials were all lower than 100, including 90 (9.3%) for the nervous system, 80 (8.3%) for the musculoskeletal system, 70 (7.3%) for blood and blood-forming organs, 42 (4.4%) for antineoplastic and immunomodulation agents, 18 (1.9%) for dermatological areas, 17 (1.8%) for anti-infectives for systemic use, and 6 (0.6%) for sensory organs (Figure 4).

**Figure 5:**
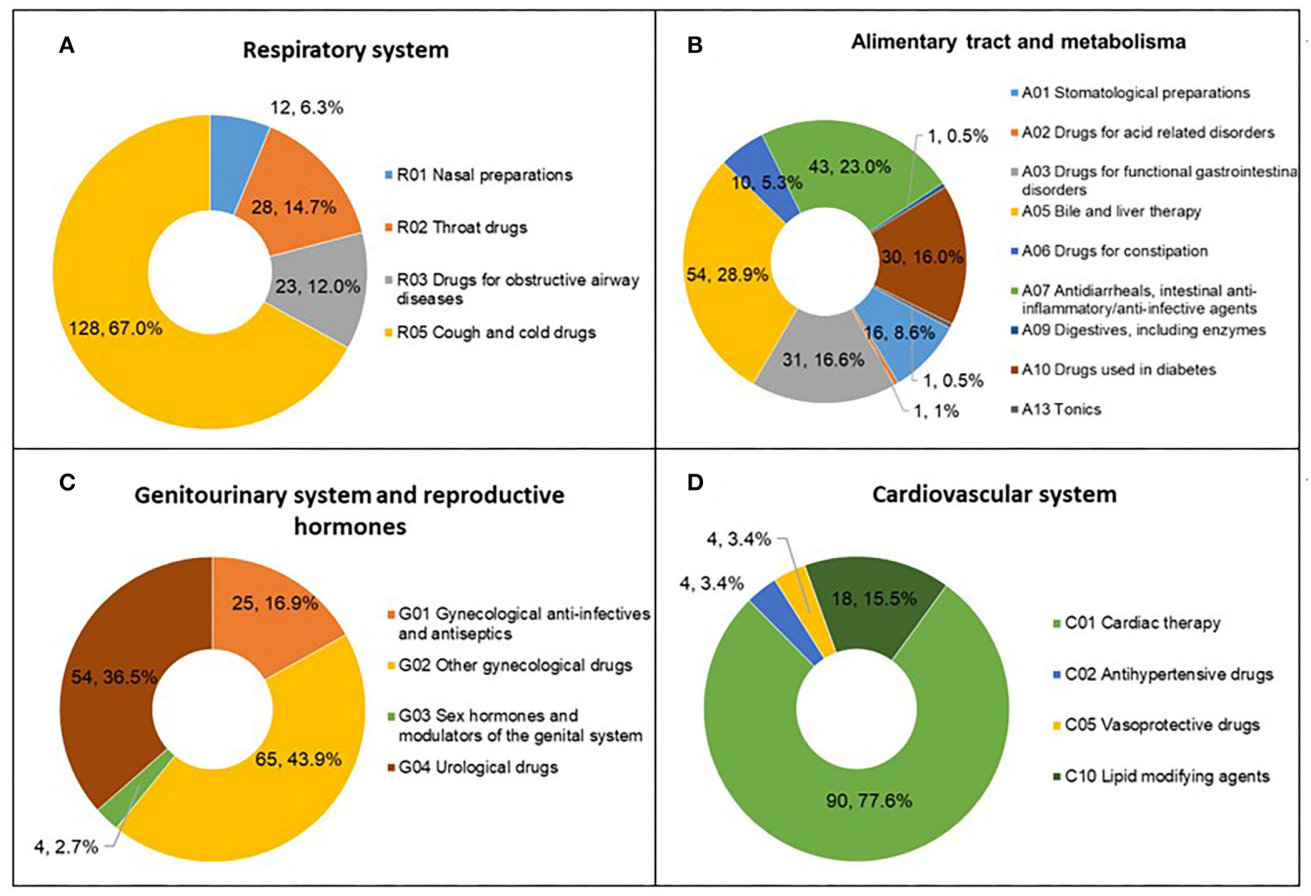
Subclass distribution of TCM trials registered from 2013 to 2021 in mainland China. **(A)** Respiratory system; **(B)** Alimentary tract and metabolism; **(C)** Genitourinary system and reproductive hormones; **(D)** Cardiovascular system.

We analyzed the subclass distribution of the top four therapy areas. Among the clinical studies of respiratory system medications, the most common trials were assigned to subclass R05 (Cough and cold drugs; 128, 67%), referring to evaluations of the safety and efficacy of cough and cold drugs (Figure 5A). For the alimentary tract and metabolism, the numbers of clinical trials assigned to A03 (Drugs for functional gastrointestinal disorders), A05 (Bile and liver therapy), A07 (Antidiarrheals, intestinal anti-inflammatory/anti-infective agents), and A10 (Drugs used in diabetes) were similar, ranging from 30 to 55 (Figure 5B); subclass A05 accounted for the largest proportion (54, 28.9%). The number of A07 trials was second only to that of A05. G02 (Other gynecological drugs) was the most studied indicator in the field of the genitourinary system and reproductive hormones, with 65 trials accounting for 43.9% of all trials (Figure 5C). C01 (Cardiac therapy) accounted for the largest proportion (90, 77.6%) in the cardiovascular system therapy area (Figure 5D), including 71 trials for angina pectoris treatment.

### Sponsors and leading clinical trial centers

**Figure 6:**
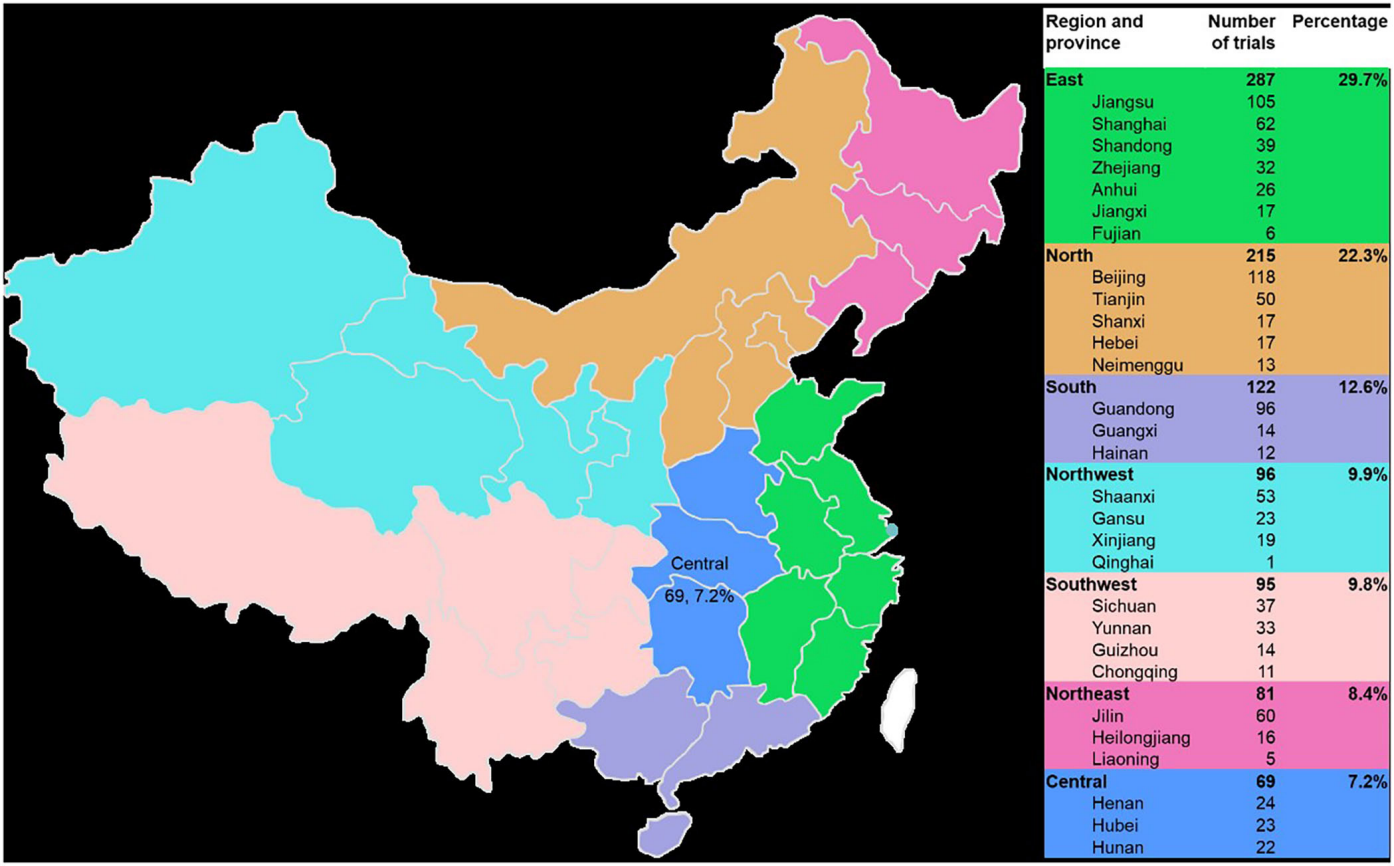
Geographical distribution of the sponsors of the 965 included TCM clinical trials from 2013 to 2021.

The 965 trials were sponsored by 610 enterprises over the last 9 years. The number of clinical trials funded by Jiangsu Kangyuan Pharmaceutical, the Institute of Medicine of Fourth Military Medical University of the Chinese People's Liberation Army, Hefei Innovative Medical Technology, and China Pharmaceutical University exceeded 10 cases, and the corresponding numbers of trials were 28, 15, 12, and 11, respectively. Large differences in the geographical distribution of these sponsors were noted. The largest numbers of sponsors were located in east China (287, 29.7%), followed by north China (215, 22.3%) and south China (122, 12.6%). The smallest numbers were in northeast China (81, 8.4%) and central China (69, 7.2%). The top five provinces in which the sponsors were located were Beijing, Jiangsu Province, Guangdong Province, Shanghai, and Jilin Province, and the corresponding numbers of trials were 116, 105, 96, 61, and 60, respectively (Figure 6).

More than 178 hospitals conducted TCM clinical trials as clinical trial units. The First Affiliated Hospital of Tianjin University of Traditional Chinese Medicine was involved in the most clinical trials among all hospitals, conducting 114 (Table 3). Furthermore, 92 hospitals only conducted one trial; most of these hospitals were directly affiliated with institutions of universities.

**Table 3:** Top 10 hospitals according to the number of TCM trials of clinical trial units from 2013 to 2021.

**Ranking**	**Trial Unit**	**Number of trials**
1	The First Affiliated Hospital of Tianjin University of Traditional Chinese Medicine	114
2	Xiyuan Hospital, Chinese Academy of Traditional Chinese Medicine	57
3	The Second Affiliated Hospital of Tianjin University of Traditional Chinese Medicine	53
4	The Second Affiliated Hospital of Guangzhou University of Chinese Medicine	52
5	Dongzhimen Hospital of Beijing University of Chinese Medicine	46
6	Shuguang Hospital Affiliated to Shanghai University of Traditional Chinese Medicine	45
7	Guang'anmen Hospital, Chinese Academy of Traditional Chinese Medicine	42
8	Beijing Traditional Chinese Medicine Hospital Affiliated to Capital Medical University	38
9	West China Hospital of Sichuan University	34
10	The First Affiliated Hospital of Hunan University of Chinese Medicine	33

### Number of new drugs approved

**Figure 7:**
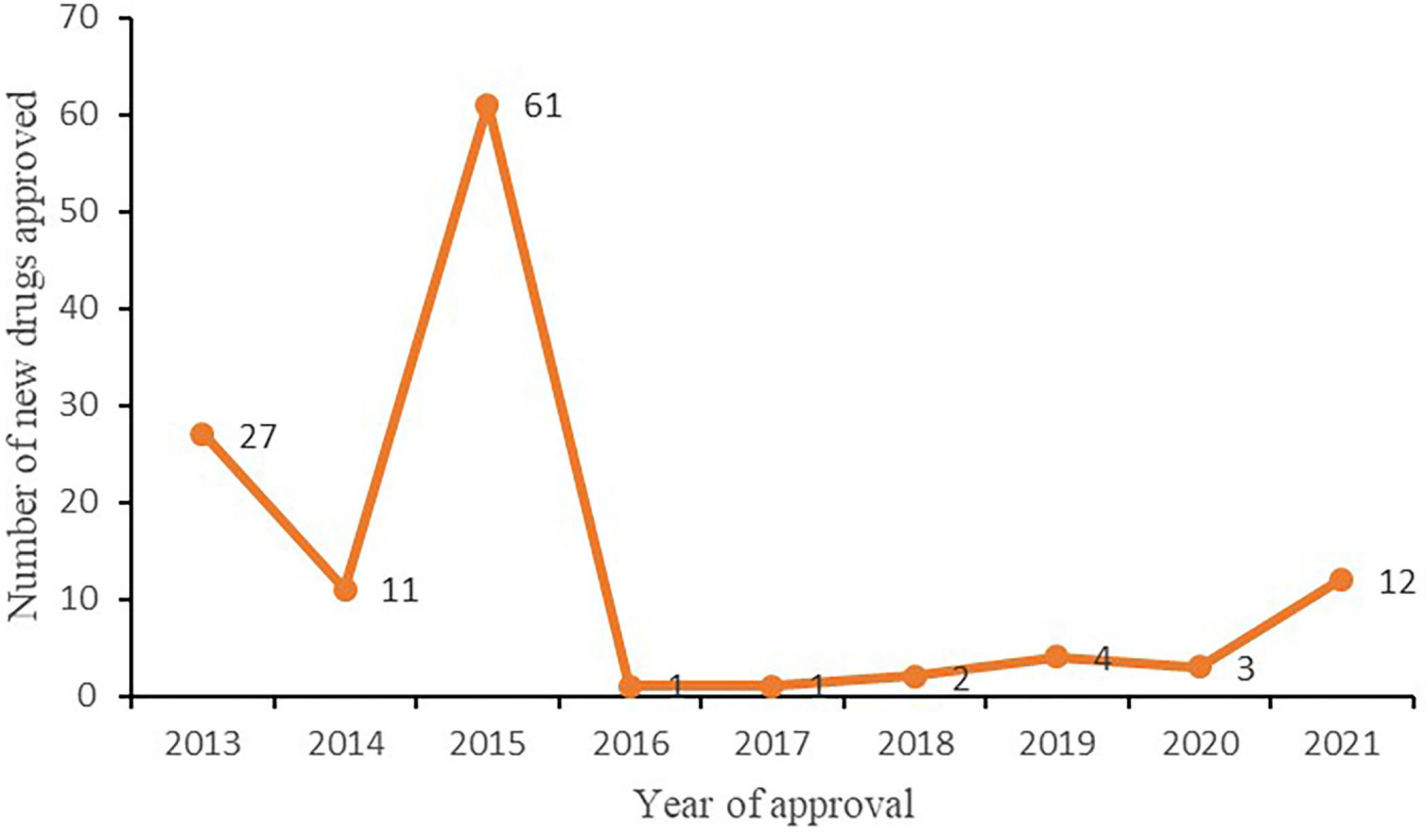
Number of new TCMs approved in mainland China from 2013 to 2021.

From 2013 to 2021, 123 new Chinese medicines were approved in mainland China, with 27, 11, 61, 2, 1, 2, 4, 3, and 12 approved each year, respectively, as illustrated in Figure 7. Among the 61 new drugs approved in 2015, 7 were classified as new drugs, 40 as drugs that change dosage form, and 14 as generic drugs. From 2016 to 2020, the number of new Chinese medicine approvals was small (Table 4). In 2021, 12 new Chinese medicines were approved, marking the highest number of TCM drug approvals in 6 years.

**Table 4:** New TCMs approved in mainland China from 2016 to 2021.

**Number**	**Approval number**	**Product name**	**Approval date**
1	Z20160001	Jinhua Qinggan Granules	2016/9/2
2	Z20170001	Danlong oral liquid	2017/8/24
3	Z20180001	Guanhuangmu granule	2018/2/2
4	Z20180002	Jinrong granule	2018/12/25
5	Z20191000	Total coumarins from Fructus Cnidii	2019/3/4
6	Z20191001	Fructus Cnidii total coumarin ointment	2019/3/4
7	Z20194049	Lishi Huayu suppository	2019/5/9
8	Z20190021	Xiao'er Jingxing Zhike granule	2019/12/16
9	Z20190022	Shaoma Zhijing granule	2019/12/18
10	Z20200001	Mulberry Twig Alkaloid	2020/3/17
11	Z20200002	Mulberry Twig Alkaloid Tablet	2020/3/17
12	Z20200003	Jingu Zhitong gel	2020/4/9
13	Z20200004	Lianhuaqingke Tablets	2020/5/12
14	C20210001	Qingfei Paidu granule	2021/3/2
15	C20210002	Huashi Baidu granule	2021/3/2
16	C20210003	Xuanfei Baidu granule	2021/3/2
17	Z20210001	Yishen Yangxin Anshen tablet	2021/9/1
18	Z20210002	Yiqi Tongqiao pill	2021/9/13
19	Z20210003	Yinqiao Qingre tablet	2021/11/9
20	Z20210004	Xuanqijiangu tablet	2021/11/24
21	Z20210005	Qizhi Yishen Capsule	2021/11/24
22	Z20210008	Jieyu Chufan capsule	2021/11/24
23	Z20210006	Kunxinning granule	2021/11/24
24	Z20210007	Huzhen Qingfeng capsule	2021/12/14
25	Z20210009	Qiruiweishu capsule	2021/12/31

Z20191000 and Z20200001 are crude drugs.

Among the new Chinese medicine drugs approved from 2016 to 2021, nine drugs are used to treat respiratory system diseases, accounting for 39.1% (9/23); six drugs are used to treat genitourinary system and reproductive hormones, accounting for 26.1% (6/23); three drugs are used to treat the nervous system, accounting for 13.0% (3/23); three drugs for alimentary tract and metabolism, accounting for 13.0% (3/23); one drug for musculoskeletal system (4.3%, 1/23); and one drug for dermatological drugs (4.3%, 1/23). Most of the new Chinese medicine approved in recent years are respiratory system, reproductive and urinary system, and sex hormones, accounting for 65.2%. In particular, Qingfei Paidu granule, Huashi Baidu granule, and Xuanfei Baidu granule were approved in 2021. These three drugs were approved to cope with the epidemic of COVID-19, which provided more choices for drugs in the clinic, trying to meet the clinical demands.

## Discussion

TCMs are types of medicinal substances prepared according to TCM theory. The main source of research and development of new TCMs is the long-term clinical practice and summary experience of TCM. The research on new-compound TCMs has been mostly based on TCM prescriptions and monitoring of the curative effect. TCM prescriptions are largely based on classic remedies, clinical experience, and folk remedies (12). The Chinese pharmacopeia is a code formulated and revised by the state to record drug standards and specifications, and it is one of the current drug standards in China (13). We have also described the efficacy of drugs with reference to pharmacopeia. However, the reference to clinical evidence is also necessary, especially because clinical research on new drugs is based on evidence. TCM has long been used to treat diseases, but its effectiveness remains controversial. In particular, NMPA-approved drugs in the early stage have not been subjected to placebo-controlled clinical trials. Rolf et al. searched the PubMed and Cochrane databases for studies analyzing the curative effect of TCM-based herbal medicines on gastrointestinal diseases. That study demonstrated a continued lack of evidence of effective treatments for gastrointestinal diseases (14). Zhang et al. analyzed the reasons why the applications for registration of new Chinese medicine were not approved from 2006 to 2008. Of the 29 varieties applied for listing, 22 (75.86%) were not approved because of their effectiveness (15). In recent years, adverse hepatotoxic reactions caused by TCM formulations have occurred from time to time. Drug-induced liver injury (DILI) has become an important reason for the failure of the research and development of TCM formulations and their withdrawal from the market. Therefore, on 12 June 2018, the guiding principle for the clinical evaluation of TCM-induced liver injury (16) issued by the NMPA will help to scientifically assess the causal relationship between the patients' liver injury and TCM. It is recommended to evaluate the causality of DILI based on the Russel Uclaf causality assessment method (RUCAM) (17–19). Melchart et al. conducted a hospital-based prospective study to determine the number of patients with liver injury after using TCM among patients without liver disease. The analysis showed that 26 of 21,470 patients (0.12%) had liver injury, and the specific manifestation was that the ALT level was higher than the normal level (20). According to the data of 6,673 million ADR reports from the China National ADR Monitoring System from 1 January 2012 to 31 December 2016, Jiabo Wang et al. found that the proportion of herb and traditional medicines-related ADR reports to the total reports was 4.5% (21). Future clinical research on new drugs should focus on conducting placebo-controlled, randomized, double-blind clinical trials to further confirm the effectiveness of TCM in treating various diseases and to evaluate adverse drug reactions.

TCM has a history of over 2,500 years. Although TCM was developed from clinical practice, with scientific progress and drug regulatory requirements, to objectively evaluate the clinical efficacy and safety of new TCMs, clinical drug trials must be conducted in accordance with GCP procedures. The investigator and sponsor play decisive roles in the overall clinical trial quality control system. Quality management includes effective trial protocol design, methods and procedures for data collection, and the collection of information necessary to make decisions in clinical trials. The contract research organization (CRO) performs its monitoring duties as required by the sponsor to ensure that the clinical trials are properly conducted and documented in accordance with the protocol. The sponsors may conduct audits in addition to routine audits to assess the conduct of clinical trials and compliance with laws and regulations. They establish procedures for auditing clinical trials as well as a trial quality management system to ensure the implementation of audit procedures in clinical trials. Although China implemented the GCP in 2003, a large gap remains between the quality of clinical research in China and that at the international level. As indicated by Wang et al., some deficiencies exist before the early clinical trials in China, such as the low proportion of randomized trials or the lack of full reporting of some critical methodological components (22, 23). Over the past 30 years, the clinical trials of new TCMs in China have made substantial advances. Numerous clinical trials of new TCMs, clinical research on protected varieties of TCMs, and the postmarket evaluation of TCMs have been completed, thereby promoting the development of the TCM industry (24). Generally, the quality of new TCMs submitted for approval has improved, but the number of approved applications is low. The number of new TCM registration applications decreased from 2015 to 2020, and the approval rate remains low (25). This is because the former State Food and Drug Administration (SFDA) announced conducting self-inspection and verification of clinical drug trial data in July 2015 to promote trial data authentication and standardization. From July 2015 to June 2017, the NMPA conducted drug clinical trial data verification on 2,033 applications for drug registration; among them, 1,316 (64.7%) were voluntarily withdrawn by the applicants. Moreover, 258 (12.7%) applications requested an exemption from clinical trials and thus did not require verification. Clinical trial data field verification was conducted on 313 drug registration applications, for which self-examination data had been submitted; of these applications, 38 were suspected of data fraud (including 16 new drug registration applications, 17 generic drug registration applications, and 5 imported drug registration applications). The NMPA denied approval for 30 of them and prompted CRO to initiate an investigation into the alleged fraud data of 11 clinical trial institutions (26). In July 2015, the NMPA issued the Announcement on Self-inspection and Verification of Drug Clinical Trial Data, which triggered enterprises to voluntarily withdraw a large number of new drug registration applications, consequently resulting in a limited number of applications and approvals for clinical trials of New Chinese medicine. Therefore, the number of applications dropped to less than 50 in 2019, which was the lowest since 2001 (27). The study by Zhou et al. showed that the disapproval rate of new Chinese medicine applied for Investigational New Drug (IND) from 2005 to 2020 reached 41.23%. Among these unapproved products, 75.88% of them had defects in pharmacological and toxicological research, of which 32.48% had errors in the selection of main pharmacodynamic experiments or major defects in experimental models, 20.58% had quality control defects such as non-standard records and difficult traceability of data, and 13.50% had defects in the design of toxicological experiments. Poor quality of basic research has become one of the main reasons hindering the development of new Chinese medicine (28). Through on-site comprehensive verification of clinical drug trial data, the quality problems of clinical trials of TCM and new drugs became more prominent. With the national large-scale verification of clinical trial data, strengthening of the supervision of clinical trial data quality, and implementation of data authentication and standardization procedures, the quality of clinical trial data has significantly improved (29). Considering the above-mentioned conditions, in recent years, our country has been trying to improve it through many measures from multiple levels of the interview map. First of all, encourage clinical trials with better policy. In 2018, National Medical Products Administration (NMPA) issued a No. 50 Announcement on Adjusting the Review and Approval Procedures of Drug Clinical Trials, which clarified that the drug clinical trials were changed from the application approval system to the implied licensing system and shortened the IND cycle. In October 2019, the Communist Party of China (CPC) Council of State Governments issued the Opinions on Promoting the Inheritance, Innovation, and Development of Traditional Chinese Medicine (TCM), emphasizing the inheritance, innovation, and development of TCM, improving the quality of TCM, and strengthening the training of TCM talents (30). On 1 July 2020, the new version of the Measures for the Administration of Drug Registration was implemented, specifying that NMPA supports the inheritance and innovation of TCM, establishes and improves the registration management system and technical evaluation system that conform to the characteristics of TCM, strengthens the quality control of TCM, and improves the level of clinical trials of TCM (31). Second, provide more strengthened technical guidance from the management level. The NMPA can refine the guiding principles for the development of TCM technology, reduce the blindness in the project development of pharmaceutical enterprises, help enterprises to select experiments more reasonably, design experiments with clear ideas, promote project development legally and normatively, improve the quality of basic research, and then improve the approval rate of IND. As evidenced by the increased number of new Chinese medicine development guidelines issued by the Center for Drug Evaluation (CDE) of NMPA in recent years, a total of 24 from 2015 to 2019 and 30 from 2020 to 2021, CDE has begun to attach importance to technical guidance in the development of Chinese medicine (32).

In terms of trial design, phase II and phase III clinical trials and randomized, double-blind, placebo, and positive drug parallel controlled trials have frequently been adopted. Regarding trial grouping, the phase II clinical trials were exploratory trials involving more than three groups; these trials accounted for 58.7%. The phase III trials generally involved two participant groups for their trial design, and the phase IV postmarket evaluation single-group design was also frequently adopted. From the previous data, increasing Phase 2 and Phase 3 trials adopt placebo control to exclude the placebo effect. In the clinical study of new traditional Chinese medicine, TCM syndromes and changes in quality of life are often judged in the form of scales. Compared with various objective laboratory indicators, non-objective indicators are more susceptible to subjective factors (33). With the continuous standardization of drug registration, the necessity of setting up placebo control has been gradually emphasized, and the proportion of placebo control clinical trials in the clinical study of new Chinese medicine has continued to increase. Before 2010, the setting rate of placebo control in clinical trials of TCM was very low (34). To more effectively evaluate the effectiveness of TCMs, currently registered clinical trials are applying the placebo control design in phase II and phase III trials more than previous clinical trials did. In terms of sample size, phase II trials are exploratory tests, and their sample sizes are typically small (35). Phase III trials are confirmatory trials, which must be determined through sample size estimation; 78.1% of the sample sizes of these trials involved more than 300 participants. The sample size of phase IV clinical trials is large, typically involving more than 2,000 participants. Randomized, double-blind, and parallel controlled approaches are the basic methodological principles applied for the objective evaluation of drug efficacy. The scientific and reasonable trial design ensures the quality of clinical trials (36). Quality is also improved through the clinical trial process itself. Zhang et al. (15) revealed that the main reasons for the rejection of TCM drug varieties submitted for approval to the drug evaluation center of the SFDA from 2006 to 2008 were problems of clinical effectiveness and a lack of standardization and authentication of research materials. In July 2015, the NMPA announced conducting self-inspection and verification of drug clinical trial data authentication and standardization. With national large-scale verification, strengthening of the quality supervision, and implementation of authentication and standardization, the quality of clinical trial data has been significantly improved.

## Conclusion

Through analysis of the registration of TCM clinical trials in China over the past 9 years, we determined that the number of clinical trials of new TCM is not high and the approval rate is relatively low. The double-blind and parallel controlled trial designs were the main designs adopted, with trial design quality exhibiting marked improvement over time.

## Author contributions

JH designed and conceived the study. YZ and JH drafted the manuscript. JY, YH, YL, CW, and HD retrieved and analyzed the data. YZ and JH revised the manuscript. All authors have read and approved the final manuscript.

## Conflict of interest

The authors declare that the research was conducted in the absence of any commercial or financial relationships that could be construed as a potential conflict of interest.

## Publisher's note

All claims expressed in this article are solely those of the authors and do not necessarily represent those of their affiliated organizations, or those of the publisher, the editors and the reviewers. Any product that may be evaluated in this article, or claim that may be made by its manufacturer, is not guaranteed or endorsed by the publisher.
